# Optimizing nitrogen fertilization in mustard through GreenSeeker-based precision agriculture: Impacts on productivity, economics, and environmental sustainability

**DOI:** 10.1371/journal.pone.0358762

**Published:** 2026-09-23

**Authors:** V.D. Meena, M.L. Dotaniya, M.D. Meena, R.S. Jat, M.K. Meena, R.L. Choudhary, H.V. Singh, H.S. Meena, B.L. Meena, V.V. Singh

**Affiliations:** ICAR-Indian Institute of Rapeseed-Mustard Research, Bharatpur, Rajasthan, India; Nuclear Science and Technology Research Institute, IRAN, ISLAMIC REPUBLIC OF

## Abstract

Excessive nitrogen (N) fertilization is a major challenge to sustainable agriculture, adversely affecting crop productivity, soil quality, and environmental health, emits a potent greenhouse gas nitrous oxide (N_2_O), and nearly 80% of sectoral emissions linked to nutrient inputs applied during crop production. The escalating use of N fertilizers further amplifies N_2_O emissions, undermining soil sustainability and contributing to environmental degradation. So, the hypothesis used behind the study is increasing nitrogen use efficiency from its current level (approx. 30–40%) would reduce the need for N fertilizer requirement, resulting in more cost-effective and environmentally sustainable production system with no yield penalty. With this objective, a field study was executed in *rabi* 2021–2024, consisting of nine N management treatments and one unfertilized treatment as control, randomized in complete block design in three replicates. To overcome this problem, first we standardized NDVI based N application and developed a Nitrogen Estimation Rate Chart (NERC) for precision N management in real time for specific set target yield. Results showed that sensor based N application enhanced growth and yield attributes, registered 22.75% seed yield enhancement with 18.7% N saving over RDF and 34.4 & 30.2% increment in NMR and BCR. This treatment augmented AE_N_ and PFP_N_ by 14.18 to 124.7% and 1.78 to 51.1%, respectively achieved higher NRE (35–69.5%). The per cent enrichment in SOC content was found superior in RDN_100_ + 2% Urea FS (52.6%) followed by RDN_100_ + 1.5% KNO_3_ FS (39.4%) statistically comparable to RDF (38.1%). The fertilizers contributed largest share of total carbon emissions, accounting for 45.6–52.3%. NDVI based N management reduced GHGs emissions by approximately 11.23% over RDF. The mean biological yield (seed + stover) unveiled positive correlation to total nitrogen (r^2^ = 98), total phosphorus (r^2^ = 95) and total potassium (r^2^ = 98) uptake. This innovative technology enables farmers to prevent excessive N fertilization, thereby reducing resource wastage and minimizing nitrous oxide emission. This technology provides insight on N need of crop in real time in right quantity.

## 1. Introduction

Rapeseed-mustard holds significant importance due to its pivotal role as a major oilseed crop with substantial potential to bridge the widening demand-supply gap of edible oils in India. Globally, it ranks as the third most important source of edible oil, following soybean and oil palm, thereby contributing substantially to nutritional security and the oilseed economy. Rapeseed-mustard crops are grown on an area of 35.95 million hectares worldwide. India ranks second in acreage (19.81%) after Canada and fourth in output (10.37%), behind China, the EU, and Canada [1]. India stands as the third-largest global producer of rapeseed-mustard, contributing over 13% to worldwide output from 2020–21–2024–25, yet its average yield of 1,461 kg/ha in 2024–25 lags at 60.7% of the global average of 2,070 kg/ha. As a pivotal oilseed crop, rapeseed-mustard ranks second in India’s agricultural landscape, trailing only soybean, and accounts for 36% of the nation’s edible oil production [2]. Overexploitation of resources coupled with reckless nutrient usage and soil fertility depletion accompanied by soil degradation, acidity and salinity causing reduction in the NUE besides enhances overall plant nutrients necessity [3]. Nitrogen is a critical element influencing chlorophyll concentration, light-use efficiency, and overall crop productivity. It governs key physiological processes in leaves, including photosynthesis, respiration, and transpiration. Besides being a common restraining nutrient for plant growth, nitrogen is a key component of numerous ecological cycles. The overuse of nitrogen fertilizers negatively impacts crop yields, degrades soil health, and harms the environment since it contributes substantially to N_2_O emissions [4–6] in addition to being an expensive agricultural input to produce and use both economically and environmentally [7]. Nitrogen losses occurring in the form of N_2_O as an active greenhouse gas significantly increases GHGs emissions and the carbon footprint too [8]. Approximately 80% of the N_2_O emission generated from agricultural sector caused by the nutrients sources applied to the soil during crop cultivation [9] and it is getting worse with more nitrogen being supplied, endangering the sustainability of the soil and environment [10]. Therefore, the biggest obstacle to preserving higher production potential, economic feasibility, environmental sustainability, and societal well-being is lack of appropriate nitrogen management [11–13].

In pursuit of higher yields, farmers often use excessive or imbalanced N rates, which can lead to soil acidification and deteriorate the soil environment, ultimately negatively affecting crop growth and yield [14,15]. According to the World Bank [16], fertilizer prices rose more than 6% year-over-year and continued to climb resulting in an 11% increase from a year prior due to continued global demand and trade restrictions. Despite this, further price fluctuations are expected, underscoring the importance of enhancing nutrient recovery efficiency via sustainable practices. It is imperative to improve the NUE from its present level, particularly of N (about 30–40%) to lessen the application of mineral fertilizers, contributing to cost-effective production and environmental preservation, while ensuring higher yields. Understanding about the demand for N, and its behaviour in soil, crop growth phase when it is critical can help in selecting nutrient management approaches which can increase the efficiency of nitrogen utilization. By optimizing resource use and reducing waste, precision agriculture empowers farming communities to make decisions that contribute to long-term environmental sustainability and food security [17,18].

The execution of IoT in agriculture can lead to substantial optimization in N use and consequent reduction in GHG emissions reduction. Sensor based application of nutrients has been shown to enhance resource efficiency significantly. Real-time data from sensors enables precise application of water, fertilizers, and pesticides, improving resource efficiency and increasing crop yields [19]. By employing precision nutrient management through IoT sensors, agricultural practices have seen a decrease in fertilizer waste by 20–40%, resulting in a 10–18% increase in nitrogen use efficiency [20,21]. For example, results from an experiment in California found that the integration of IoT sensors in nutrient application reduced fertilizer expenditures by approximately 25% while achieving yields similar to or better than traditional methods [21]. Data suggest that precision agriculture not only mitigates costs related to over-fertilization but also reduces environmental impact of excess nutrient runoff. In an experiment results showed that IoT sensors can help in saving 28–39% of irrigation water and 9–80% of fertilizers based on fertilizers type considered, thus importantly contributing to reduce GHG emissions being nitrous fertilizers application a major contributor to total agricultural GHG emissions [22]. In addition to posing environmental concerns, excess nitrogen application increases production costs and can lead to yield reductions. Current management practices do not consistently lead to increased yields or facilitate reduced nitrogen fertilizer application rates. In situations where nitrate leaching does not occur or fertilizer N application rates are adequate, nitrogen losses may not constrain yield, and therefore these management practices may offer limited benefit. Since fertilizer decisions are generally made without knowing how the season will progress, it is important to use practices that maximize nitrogen use efficiency and prevent nitrate accumulation in the root zone. Optimal N rates are site-specific and must be estimated based on expected yield.

In response to these challenges, the application of precision agriculture through IoT systems could represent a promising strategy for better nitrogen management while maintaining or improving productivity and sustainability. Sensor based N application; an innovative technology can facilitate precise N administration by enabling real-time monitoring and management by optimizing fertilizer N. The study aimed to estimate precise N amount in real time with respect to targeted yield, delivering N when crop require it most and to assess its effects on input savings, resource use efficiencies and productivity of mustard, providing more comprehensive insights than those currently available in existing literature.

## 2. Materials and methods

### 2.1. *Site description and soil type*

In this study we conducted a field experiment at research farm of ICAR-Indian Institute of Rapeseed-Mustard Research, Bharatpur with latitude and longitude of 27°12’8.9“ N and 77°27’18.8” E at 178.4 m above MSL altitude during winter season of 2021−22–2024−25 using *Brassica juncea* L var. DRMR 2017−15 as test crop. The experimental site fall under subtropical, semi-arid climate, receiving approximately 650 mm of annual rainfall, with 85–90% of which received during July to September. The soil texture was categorized as clay loam has low nitrogen (126.3 kg ha^-1^) and organic carbon (2.4 g kg^-1^), medium in extractable phosphorus (17.2 kg ha^-1^) and exchangeable potassium (1.0 N NH4OAc, 149.3 kg ha^-1^) with alkaline soil pH (8.3) having an EC of 1.30 dS m^-1^.

### 2.2. *Experimental setup and crop husbandry*

For this, first we standardized the NDVI value for nitrogen fertilization in mustard crop and after that developed a Nitrogen Estimation Rate Chart (NERC) for accurate determination of nitrogen amount to be applied as and when required by the crop at various phonological phases. The field study was arranged in randomized complete block design with three replications consisting of ten treatments. The seed treatment was done with Apron 35 SD @ 6 g/kg seed and sown by using seed drill in the second fortnight of October every year. The seeds were placed in the furrow at 2–3 cm below in depth with 45 cm line to line (row) and 15 cm distance between the plants using seed @ 3.5 kg ha^-1^. Uniform application of recommended dose of fertilizers *viz*. N, P_2_O_5_, K_2_O, S and B were provided to the crop at the rate of 80-40-40-40-1 kg ha^-1^, respectively through urea, SSP, MOP, sulphur and borax. Half of the nitrogen and full doses of PKSB were applied as a basal to respective plot at sowing. The remaining half of nitrogen (50%) was top dressed at the time of first irrigation, i.e., 30–35 days after sowing in accordance with specific treatments using different N management techniques designed to satisfy the crop’s unique nutritional requirement. Excess plants were removed (at 18–21 DAS) to maintain optimum spacing between the plants. Necessary inter-culture operations were performed throughout the crop-growing period in each treatment plot. Crop was harvested manually at physiological maturity in the month of March-April and threshed after 3–4 days of drying and weighed seed yield from each experimental unit independently. Data on growth and yield characteristics *viz*. plant height, number of branches, siliquae number, seeds per siliqua, weight of 1000 seed, economic and biomass yields were measured at harvest.

### 2.3. *GreenSeeker hand held crop sensor (GS)*

The GS device (Trimble Pvt. Ltd., USA) is a cost-effective, user-friendly handheld measurement tool widely used to assess plant health and vigor for precise nutrient management. The device provides instant readings on plant status, which are used to make objective decisions regarding the amount of nitrogen fertilizer to be applied. This enables more precise nitrogen use, benefiting both farmers and the ecosystem. The sensor emits brief bursts of red and infrared light and measures the amount reflected back from the green plant canopy. The measured values are displayed on an LCD screen, ranging from 0.00 to 0.99, and are expressed as NDVI (Normalized Difference Vegetation Index) readings. The reflectance of incident light from plants depends on leaf tissue structure and chlorophyll content. Chlorophyll in the palisade layer absorbs approximately 70–90% of incident visible light (400–720 nm), whereas mesophyll tissues reflect about 60% of incident near-infrared (NIR) radiation (720–1300 nm) [23].

### 2.4. *Development, calibration and validation of NDVI*

For calibration of NDVI values, first we have developed separate N-rich strips along with the experimental plots by applying 75%, 100%, 125%, 150%, 200%, 250% and 300% of RDN (recommended dose of N @ 80 kg/ha) as 60, 80, 120, 160, 200 and 240 kg N ha ^−^ ¹ in split doses to maintain sufficient nitrogen availability throughout the crop growing period and NDVI values were recorded at 7–10 days intervals. A constant value of NDVI recorded from the different N-rich strips was standardized as optimum NDVI value for achieving sustainable mustard yield with higher nutrient use efficiency. Concurrently, the validation of the results was executed at different locations for two years. On the basis of recorded data NDVI was computed using the following equation:


$$\begin{array}{c} NDVI\;=\frac{FNIR-FRed\;}{FNIR+FRed\;} \end{array}$$

Where, F_NIR_ and F_Red_ indicate the proportions of emitted near-infrared and red radiation reflected from the sensed area, respectively.

The assumption of a constant yield coefficient can be justified by testing whether plant nitrogen uptake is approximately proportional to biomass production. Let the yield coefficient be


$$\begin{array}{c} YN=\frac{\Delta B}{\Delta N} \end{array}$$

Where B is plant biomass and N is plant nitrogen uptake. A constant YN implies that the relationship between biomass accumulation and nitrogen uptake remains approximately linear.

### 2.5. *Carbon footprint*

Carbon footprint was estimated to assess the greenhouse gas (GHG) emission potential of different nitrogen management options. It was calculated as the total GHG emissions produced during the crop growing season and expressed as carbon dioxide equivalents (CO_2_-e) [24]. Carbon dioxide emissions associated with various agricultural inputs were estimated using the carbon emission factors reported by Deng [25]; West and Marland [26]; Lal [27]; Wang et al. [28] and expressed in kg CO_2_-eq ha ^−^ ¹. The total CO_2_ emissions were obtained by summing the emissions from all input sources.


$$\begin{array}{c} Carbon\;footpriniting\;=\frac{Carbon\;emmission\;\left(kg\;CE\;ha^{-\text{1}}\right)}{Mustard\;seed\;yield\;\left(kg\;ha^{-\text{1}}\right)} \end{array}$$

$$\begin{array}{c} N_{\text{2}}O\;emmission\;\left(kg\;N_{\text{2}}O\;year^{-\text{1}}\right)=Total\;added\;N\;\left(kg\;ha^{-\text{1}}\right)\;\times \;EF\;\times \;\frac{\text{4}\text{4}}{\text{2}\text{8}} \end{array}$$

Where, the ratio of atomic weights of N_2_O to N_2_ (44/28 = 1.571) determines the conversion of N to N_2_O, or the quantity of N_2_O released per unit of N use. The equation given by Tubiello et al. [29] represents the kg N_2_O–N emitted per kg N input obtained by multiplying the emission factor (0.01) for N_2_O emissions with the amount of N applied from fertilizer.

The effect of different nitrogen management options was evaluated by estimating the carbon footprint on an ecological, area-based (spatial), and yield-scaled basis. The ecology-based spatial carbon footprint represents the total CO_2_ and N_2_O emissions generated during the crop growth period, expressed as carbon dioxide equivalents (CO_2_-e) [24]. Methane (CH_4_) is produced under anaerobic soil conditions when microorganisms lack oxygen as an electron acceptor and CO_2_ is reduced to CH_4_. Therefore, only CO_2_ and N_2_O emissions were considered in the carbon footprint estimation. In addition, no on-farm burning of crop residues or other agricultural biomass was seen during the experimental period. The emitted CO_2_ and N_2_O were converted to CO_2_ equivalents on multiplication of global warming potentials (GWPs) of 1 and 298 [30]. The assessment included greenhouse gas emissions associated with the production and use of all agricultural inputs applied during the experiment.

The GWP estimate made with equation:


$$\begin{array}{c} GWP=(emitted\;N_{\text{2}})\;\times \;\text{2}\text{9}\text{8}+emitted\;CO_{\text{2}} \end{array}$$

The CFs was worked out by using equation as given by Pandey and Agrawal [31].


$$\begin{array}{c} CFs\;=\sum_{i=\text{1}}^{n}GWP\;(kg\;CO_{\text{2}}-e/ha) \end{array}$$

Here, CFs refers to carbon-footprint on unit area basis


$$\begin{array}{c} CFy\;=\frac{CFs}{Biological\;yield}\;(kg\;CO_{\text{2}}-e/t) \end{array}$$

Where, CFy denotes per unit yield of CFs (yield-scaled carbon footprint), mustard biological yield is in t ha^−1^.

### 2.6. *Monetary analysis*

Every treatment’s economic viability was calculated following the current market prices of the input items that were employed, such as fertilizer, seeds, and other supplies in the production. The overall cost of production included fuel for machines, labour costs for irrigation, seed, nutrients, and harvesting. The following equations were used to calculate the benefit-cost ratio (BCR) [32].


$$\begin{array}{c} Cost\;of\;cultivation\;(CC)\;(US\$\;ha^{-\text{1}})=Input\;cost\;(US\$\;ha^{-\text{1}})+Labour\;cost\;(US\$\;ha^{-\text{1}}) \end{array}$$

$$\begin{array}{c} Net\;monetary\;return\;(NMR)\;(US\$\;ha^{-\text{1}})=Gross\;return\;(US\$\;ha^{-\text{1}})-CC\;(US\$\;ha^{-\text{1}}) \end{array}$$

$$\begin{array}{c} Gross\;monetary\;return\;(GMR)\left(US\$\;ha^{-\text{1}}\right)=Economic\;yield\;\left(kg\;ha^{-\text{1}}\right)\;x\;Minimum\;support\;price\;(MSP)\;(per\;kg) \end{array}$$

$$\begin{array}{c} BCR\;(US\$\;ha^{-\text{1}})=GMR\;(US\$\;ha^{-\text{1}})/CC\;(US\$\;ha^{-\text{1}}) \end{array}$$

### 2.7. *Statistical analysis*

The SAS 9.3 statistical software (IBM ver.23) was used to statistically analyze the data in order to determine treatments significance. An ANOVA (analysis of variance) was accomplished to establish the significance of treatments on various observations from four sequential years and averaged to get the mean. The mean effect of treatments were distinguished using DMRT [33] at *p* = 0.05 level of significance.

## 3. Results

Data recorded during different years (2021–2024) were pooled to interpret, writing results and discussions and to draw concrete conclusion. The results and discussions section were written on the basis of pooled data.

### 3.1. *NDVI based N fertilizer rate estimation*

**Fig 1:**
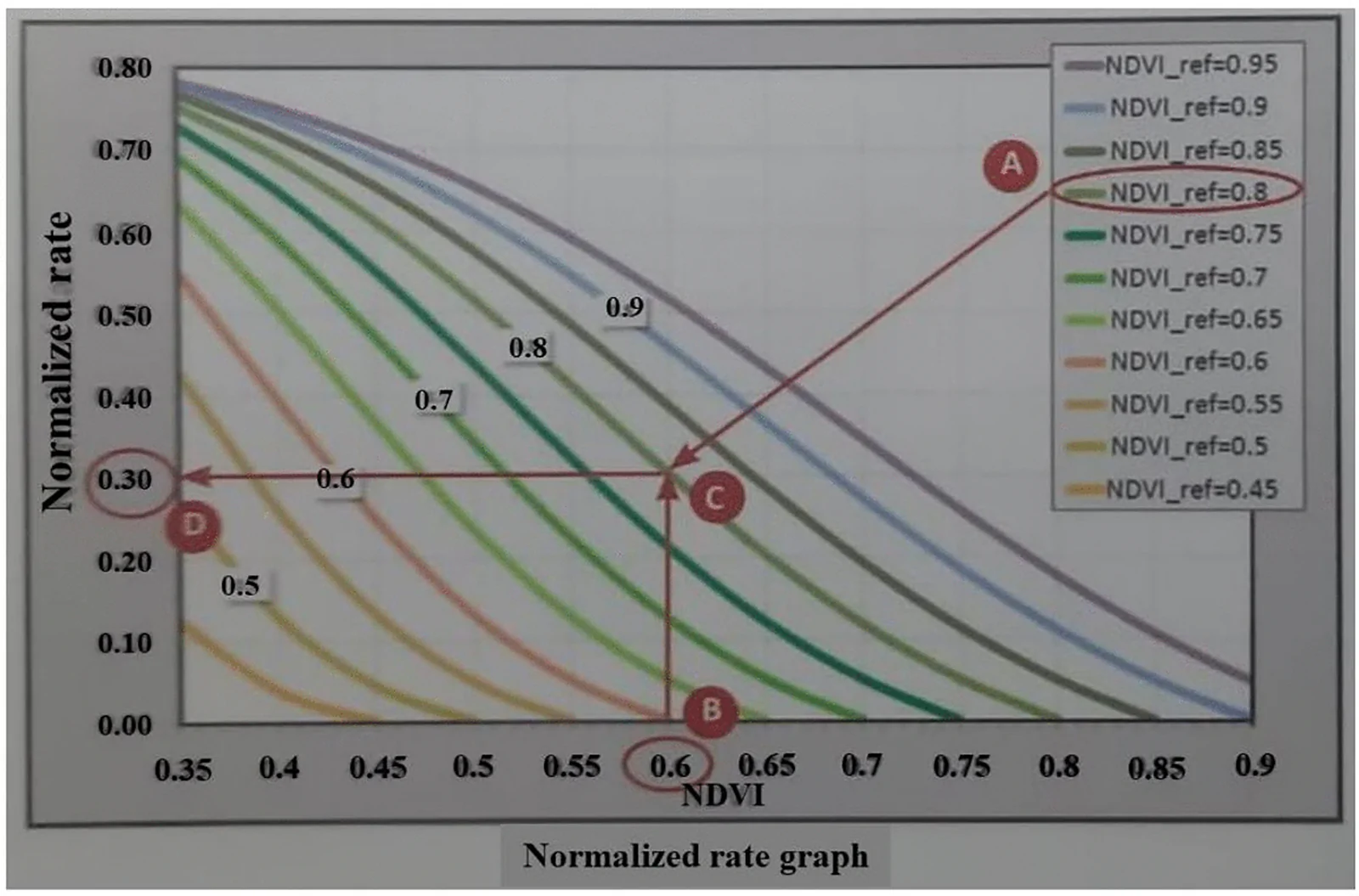
Normalized rate estimation chart (Trimble).

For estimating N requirement of mustard at different growth phases using GS crop sensor, the following steps to be followed:

i) First of all, we have to measure the NDVI value from reference area that is nitrogen rich area (N strip plots) named as NDVIref (A); ii) Then measure NDVI value from non-reference area named as NDVIfp (B); iii) Note down the intersect value of A and B form the given chart as (C); iv) Find out the normalized rate indicated in front of the intersect point from the chart named as (D) (Fig 1); v) At the end, multiplication of the normalized rate with the crop factor will arrive to the amount of N to be applied at a time or at that particular crop growth stage in the field condition.

### 3.2. *Crop factor calculation (CFC) for specific targeted yield*

Crop factor was derived for achieving the targeted mustard seed yield by taking the average of total N content in the plant as 2.10% with 55% nitrogen use efficiency (Table 1). Based on the NDVI value, we have developed the N estimation rate chart (NERC) at different normalized rate for different targeted yield (Table 2). The NDVI value from the reference area (N rich strip) was standardized as 0.80 for mustard during experimentation.

**Table 1:** Calculation of crop factor (CFC) at different targeted yield of mustard.

Mustard (% N = 2.10)	Targeted yield (t ha^−1^)							
	1.0	2.0	2.5	3.0	3.5	4.0	4.5	5.0
Crop factor	38	76	95	114	134	152	172	191

**Table 2:** N application rate chart developed (NERC) for set targeted yield based on the NDVI value in mustard.

NDVIref	NDVIfp	Normalized rate	Nitrogen requirement (kg ha^−1^) Targeted yield (t ha^−1^)							
			1.0	2.0	2.5	3.0	3.5	4.0	4.5	5.0
0.80	0.70	0.1	4.6	9.1	11.4	13.7	16.0	18.2	17.2	19.1
0.80	0.65	0.2	7.6	15.2	19.0	22.8	26.6	30.4	34.4	38.2
0.80	0.60	0.3	11.4	22.8	28.5	34.2	39.9	45.6	51.6	57.3
0.80	0.55	0.4	15.2	30.4	38.0	45.6	53.2	60.8	68.8	76.4
0.80	0.50	0.5	19.0	38.0	47.5	57.0	66.5	76.0	86	95.5
0.80	0.45	0.6	22.8	45.6	57.0	68.4	79.8	91.2	103.2	114.6
0.80	0.40	0.7	25.8	51.7	64.6	77.5	90.4	103.4	120.4	133.7

Abbreviations: NDVIref: normalized difference vegetation index from reference area; NDVIfp: normalized difference vegetation index from non-reference area (treatment plot)

For understanding the equations we take the example as suppose our mustard target yield is 3 t/ha and the crop factor for 3.0 t/ha targeted yield is 114 (Table 2) and assuming that the NDVI value of the non-reference area (treatment plot) is 0.60 then the normalized rate from the intersect of NDVIref (A) and then NDVIfp (B) will arrive to 0.30. From these above values, we get the estimated N requirement for set targeted yield is 34.2 kg/ha by multiplying the normalized rate with respective crop factor. From this calculation, it makes clear that for achieving mustard yield of 3.0 t/ha, we have to apply 34.2 kg of nitrogen as topdressing at that particular time or crop growth phase.

#### Quantitative validation metrics for NERC chart.

We quantified the internal consistency of the normalized-rate values by treating the 1.0 t/ha value for each NDVIfp as the reference and testing whether the values at higher target yields follow the expected proportional relationship:


$$\begin{array}{c} R_{\text{expected}}=R_{\frac{1t}{ha}}\times Y \end{array}$$

In our study, we evaluated all 56 rate yield combinations. The proposed normalized-rate matrix demonstrated strong internal consistency with the expected proportional relationship between targeted yield and normalized rate, with an RMSE of 1.12, MAE of 0.39, and R^2^ of 0.9988, i.e., 99.88% (n = 56). These data shows very high internal consistency with the expected linear scaling of normalized rate with targeted yield. However, independent field or experimental observations are required for external validation of the proposed rate recommendations.

### 3.3. *Growth, yield attributes and monetary analysis*

Application of nitrogen under various treatments significantly affected the growth attributes *viz*. primary and secondary branches per plant, siliqua length except plant height and yield attributes such as siliqua per plant, seed per siliqua, 1000 seed weight, seed and stover yield (Table 3). The greater improvement with respect to growth attributes were seen under GS based N application as compare to other N-application methods followed by LCC based nitrogen management. The NDVI based N application achieved per cent increase of 8.6, 12.7, 5.71 & 11.2% and 24.6, 39.3, 21.7 & 15.7%, respectively in primary and secondary branches/plant, siliqua length and seeds per siliqua over the recommended management practice and control.

**Table 3:** Yields attributes, yield and monetary analysis (profitability) of *Brassica juncea* L. as influenced by different nitrogen management modules (Mean data of four years).

Treatments	Growth attributes				Yield attributes					HI (%)	NMR (US$/ha)	BCR
					Siliquae/ plant (no.)	Seed/ siliquae (no.)	1000 seed weight (g)	Seed yield (kg ha^-1^)	Stover yield (kg ha^-1^)			
	PH	PB	SB	SL								
RDN_100_ (50% N basal + 50% N TD) + 2% Urea FS	2.01^a^	5.88^b^	11.65^bc^	5.01^b^	389^d^	16.12^a^	5.58^ab^	2376^c^	6916^b^	0.26^a^	1048^c^	2.63^ab^
RDN_100_ (50% N basal + 50% N TD) + 2% NCU FS	2.01^a^	5.98^b^	11.90^bc^	5.05^b^	395^c^	16.27^a^	5.63^a^	2471^b^	6999^a^	0.26^a^	1124^b^	2.75^ab^
RDN_100_ (50% N basal + 50% N TD) + 1.5% KNO_3_ FS	1.98^a^	5.81^b^	11.38^bc^	4.89^b^	378^ef^	15.94^a^	5.30^a^	2280^e^	6747^c^	0.25^a^	910^de^	2.47^b^
RDN_75_ (50% N basal + 25% N TD) + 2% Urea FS	1.98^a^	5.73^c^	10.99^c^	4.84^b^	362^g^	15.84^a^	5.39^ab^	2244^f^	6569^e^	0.25^a^	944^e^	2.47^b^
RDN_75_ (50% N basal + 25% N TD) + 2% NCU FS	1.97^a^	5.75^c^	11.26^bc^	4.90^b^	374^f^	15.98^a^	5.60^ab^	2323^d^	6496^e^	0.26^a^	1028^d^	2.57^ab^
RDN_75_ (50% N basal + 25% N TD) + 1.5% KNO_3_ FS	1.98^a^	5.53^c^	10.39 cd	4.77^b^	354^h^	15.67^a^	5.29^ab^	2171^g^	6353^g^	0.25^a^	845^f^	2.35^bc^
RDN – Green seeker	2.00^a^	6.34^a^	12.72^a^	5.24^a^	432^a^	16.64^a^	5.64^a^	2681^a^	6940^a^	0.28^a^	1276^a^	3.10^a^
RDN – LCC	2.02^a^	6.10^b^	12.44^b^	5.01^b^	407^b^	16.25^a^	5.72^a^	2464^b^	6783^d^	0.27^a^	1187^b^	2.75^ab^
RDF	2.00^a^	5.84^c^	11.29^bc^	4.71^b^	381^e^	15.74^a^	5.44^ab^	2184^g^	6460^f^	0.25^a^	949^f^	2.38^b^
Control	1.90^a^	5.09^d^	9.13^d^	4.53^c^	321^i^	13.67^b^	5.04^b^	1577^h^	4902^h^	0.24^a^	487^g^	1.75^c^

Different letters in each column represent significant differences at p ≤ 0.05 level based on Duncan’s multiple range test

Abbreviations: RDN: recommended dose of nitrogen, TD: top dressing; FS: foliar spray, NCU: neem coated urea, RDF: recommended dose of fertilizer, LCC: leaf colour chart, PH: plant height, PB: primary branches, SB: secondary branches, SL: siliqua length, HI: harvest index, NMR: net monetary returns, BCR: benefit cost ratio.

Also, the treatment registered substantial effect on economic and biomass yield and subsequently on their economic feasibility. The per cent increment in mean economic yield (seed) over the four years was lies between the 2.77 to 22.75% over the recommended practice except in treatment RDN_75_ + 1.5% KNO_3_ FS, where slightly decline was recorded (−0.59%). The highest seed yield enhancement (22.75%) due to variable N management modules was observed on N application in GS whereas, maximum decline in economic yield was noticed under control (−22.78%) in the absence of nutrient application including nitrogen. Among N foliar spray of urea, NCU and KNO_3_ at 2.0 or 1.5%, N at RDN_100_ was marginally better than RDN_75._ Similar trend of results were also seen in stover yield and harvest index amongst treatments, but it was found non-significant. However, highest reduction in the dry biomass was observed in control treatment (−20.88%) over the RDF. The ratio of seed yield to biological yield was ranged in between 0.24 to 0.28 under various treatments. Similarly, the results pertaining to treatment economic feasibility showed maximum NMR (net monetary returns) and BCR (benefit-cost ratio) in the treatment where N was administrated through GreenSeeker. The per cent increase in NMR and BCR under sensor guided N application was 34.4 and 30.2% over recommended practices followed by RDN_100_ + 2% Urea FS and RDN-LCC (Table 3), whereas, control treatment fetched lowest values of NMR and BCR amongst all N management modules. Foliar spray of nitrogen through NCU @ 2% performed better in comparison to other N foliar spray sources (urea and KNO_3_) at both N levels.

### 3.4. *Per cent N saving over RDF*

The suggested dosage of N application for mustard crop is 80 kg N/ha, in which, half of the recommended N, i.e., 50% was applied at the time of sowing as basal, and remaining half N to be topdressed at first irrigation (30–35 DAS). Besides recommended N dosage, more or less amount of N was also applied under various treatments to the crop (Table 4). Data in the table showed that, about 18.7% N was saved over RDF, when N management was made on the basis of NDVI value in comparison to other N treatment followed by LCC based N management (10% N saving). Whereas, additional N were administrated on foliar spray via urea/NCU or KNO_3_ at RDN_100_. However, the maximum saving of N were observed at RDN_75_ in comparison to RDN_100_ because of applying of only 75% of RDN. The total N applied to the crop varies from 65 to 85.52 kg N/ha based on the treatment requirement.

**Table 4:** Total nitrogen application v/s crop nutrient removal (NPK) variations under different nitrogen management modules (Mean data of four years).

Treatments	N application				% N saving over RDF	TNU (kg ha^−1^)	TPU (kg ha^−1^)	TKU (kg ha^−1^)	% change in nutrient removal over RDF		
	Basal	Top dressing	FS	Total N					TNU	TPU	TKU
RDN_100_ (50% N basal + 50% N TD) + 2% Urea FS	40	40	5.52	85.52	−6.90	79.16^c^	42.03 cd	148.29^c^	10.5	4.8	14.7
RDN_100_ (50% N basal + 50% N TD) + 2% NCU FS	40	40	5.52	85.52	−6.90	82.82^b^	45.12^b^	152.61^b^	15.6	12.5	18.0
RDN_100_ (50% N basal + 50% N TD) + 1.5% KNO_3_ FS	40	40	1.17	81.17	−1.46	75.59^de^	38.81^e^	139.93^d^	5.5	−3.3	8.2
RDN_75_ (50% N basal + 25% N TD) + 2% Urea FS	40	20	5.52	65.52	18.10	73.95^ef^	39.21^e^	140.07^d^	3.2	−2.3	8.3
RDN_75_ (50% N basal + 25% N TD) + 2% NCU FS	40	20	5.52	65.52	18.10	77.52 cd	40.61d^e^	140.82^d^	8.2	1.2	8.9
RDN_75_ (50% N basal + 25% N TD) + 1.5% KNO_3_ FS	40	20	1.17	61.17	23.54	68.64^g^	32.98^f^	129.31^e^	−4.2	−17.8	0.0
RDN – Green seeker	40	25	–	65	18.75	88.83^a^	48.56^a^	159.45^a^	24.0	21.0	23.3
RDN – LCC	40	32	–	72	10.00	82.38^b^	43.88^bc^	147.78^c^	14.9	9.4	14.3
RDF	40	40	–	80	0.00	71.66^f^	40.12^de^	129.31^e^	0.0	0.0	0.0
Control	–	–	–	–	–	43.66^h^	21.48^g^	89.91^f^	−39.1	−46.5	−30.5

Different letters in each column represent significant differences at p ≤ 0.05 level based on Duncan’s multiple range test

Abbreviations: RDN: recommended dose of nitrogen, TD: top dressing; FS: foliar spray, NCU: neem coated urea, RDF: recommended dose of fertilizer, LCC: leaf colour chart, TNU: total nitrogen uptake, TPU: total phosphorus uptake, TKU: total potassium uptake.

### 3.5. *Nutrient use efficiency indicators*

#### 3.5.1. *AE*_*N,*_ *PFP*_*N*_ *and recovery efficiency of NPK.*

**Fig 2:**
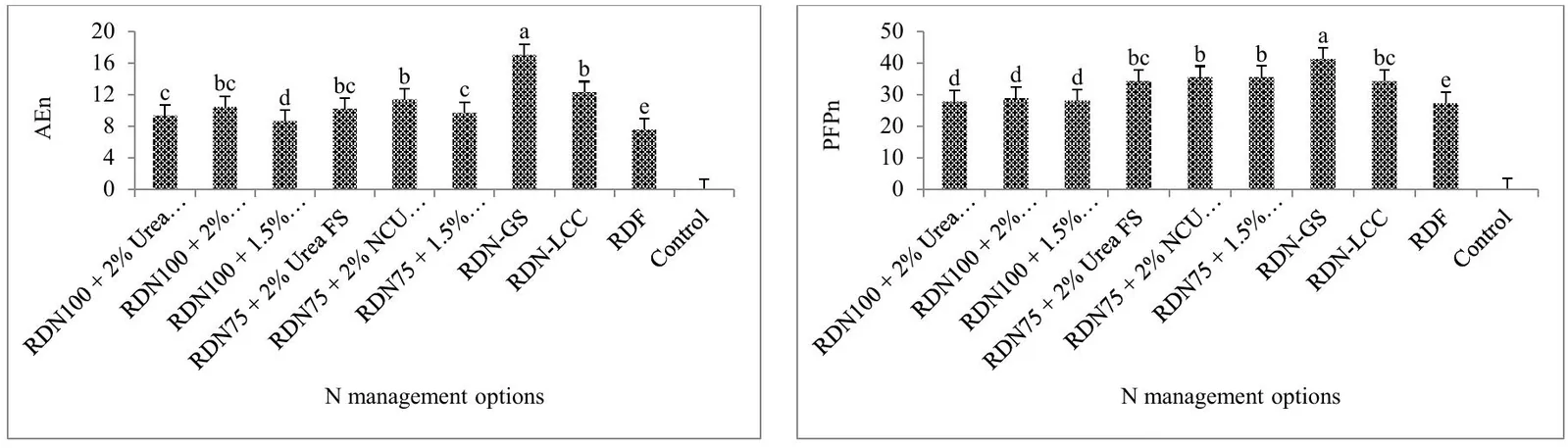
Effect of N management options on a) agronomic efficiency and b) partial factor productivity in mustard production (Mean data of four years).

**Fig 3:**
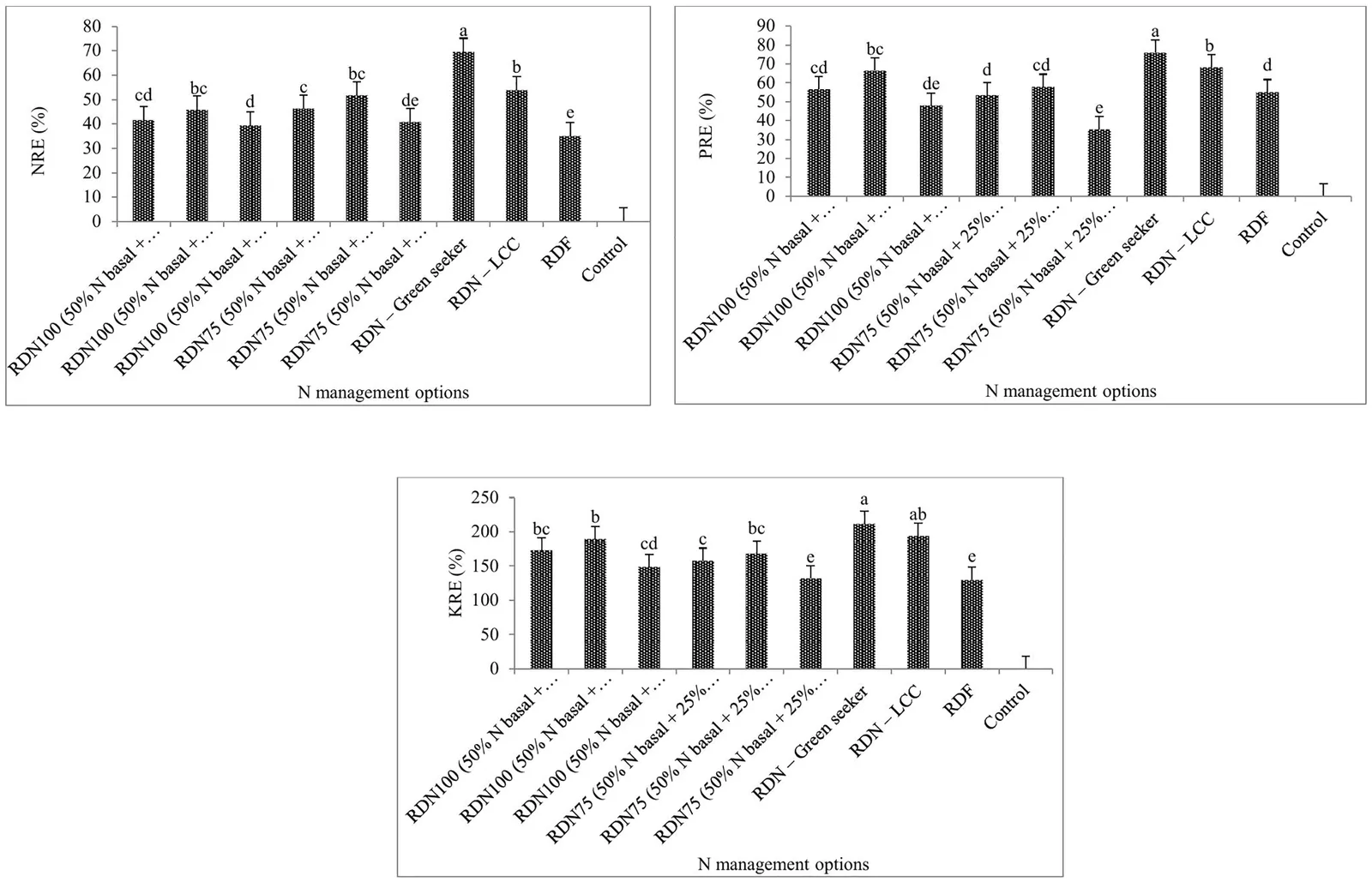
Recovery efficiency of NPK as NRE, PRE and KRE affected by various N source and their levels in mustard (Mean data of four years).

Various nutrient efficiency indices were computed and presented in the Fig 2. The contribution of fertilizer N towards yield, compared to non-fertilized control (AE_N_-agronomic efficiency) and values of yield produced per unit use of nitrogen, i.e., partial factor productivity (PFP_N_) was accomplished superior under N application through sensor based GS over rest of the N management options. The widerper cent variations in the pooled data of AE_N_ and PFP_N_ were observed which ranges from 14.18 to 124.7% and 1.78 to 51.1%, respectively among the N management treatments. The treatment LCC and RDN_75_ with 2% NCU FS was next higher after GS with respect to AE_N_ (62.4% & 50.1%), whereas, the values for PFP_N_ was higher under GS (51.08%) subsequently foliar spray of N through urea, NCU and KNO_3_ at RDN_75_ over the RDN_100_. The N management treatments with RDN_100_ were performed poorly over the recommended practice (RDF). Moreover, the per cent recovery efficiency of N widely ranged from 35–69.5% amongst N management options (Fig 3). Nutrient recovery by the crop (seed + stover) was found maximum when N applied using GS which accredited to the retaining of significantly higher NRE or NUE (69.5% average of four years) as compared to RDF (35.0%) as lowest followed by LCC (53.8%). Due to reduced amount of N at RDN_75_, the NRE percentages of these treatments were marginally greater than the treatments at RDN_100_. Similar trend of results in nutrient absorption of P and K (PRE & KRE) was noticed and ranged from 28.7–67.7 and 100.8–176.1%, respectively. The crop responses were significantly improved due to various nutrient application methods. The values of NRE, PRE and KRE were observed greater under GS guided N management followed by LCC based N module and the lesser values were recorded under RDF. Nitrogen administration at recommended level (RDN_100_) registered marginally lesser NRE (nitrogen recovery efficiency) as against to their lower level at RDN_75_.

#### 3.5.2. *Crop nutrient removal (TNU, TPU, TKU).*

Cumulative nutrient absorption data indicated that mustard is a highly nutrient-demanding crop. The significant variations were observed in the plant nutrient uptake among the various N management options over the four year studies. The mean data on total N, P and K uptake were varied with nutrient doses, levels and application method by which these applied. The highest total uptake (seed + stover) of N, P and K was estimated under GS guided N management (24.0, 21.0 & 23.3% higher) over the recommended practice (RDF) followed by LCC-N (14.9, 9.4 & 14.3% higher) (Table 4). The significant decline in the values of TNU, TPU and TKU (−39.1, −46.5 & −30.5%) were seen under control treatment in the absence of N and other nutrient application. The total phosphorus absorption was more affected compared to N and K uptake. Among N foliar spray, N administration through NCU was given better results in relation to urea and KNO_3_ at RDN_100_ than RDN_75_ whereas other treatments were non-significant and not performed to the satisfactory level.

**Fig 4:**
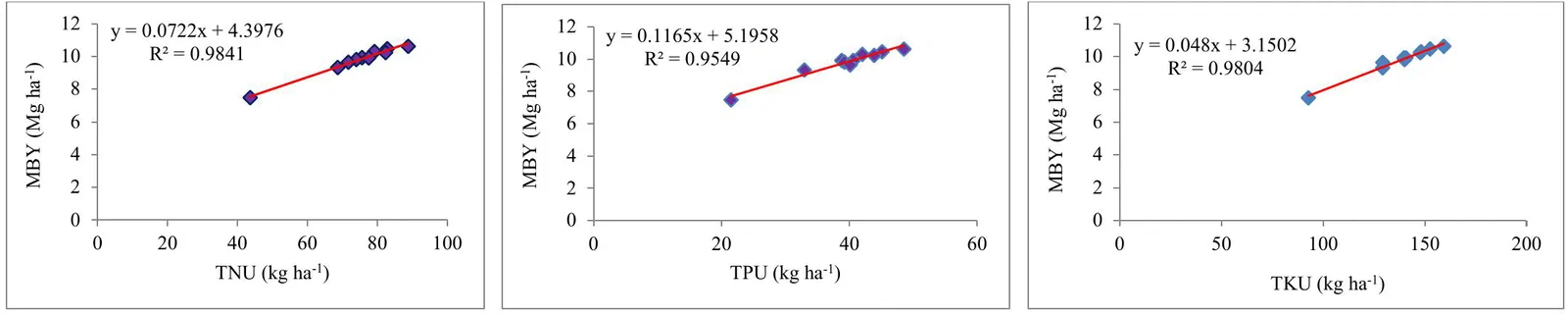
Linear regression lines showing relationship between mustard biological yield (MBY) with total uptake of (a) nitrogen (TNU (b) phosphorus (TPU), and), potassium (TKU) in mustard.

The assumption of a constant nitrogen yield coefficient was supported by the measured relationship between total nitrogen uptake (TNU) and maximum biomass yield (MBY) (Fig 4). Across the ten treatments, MBY exhibited a strong positive linear relationship with TNU (R^2^ = 0.9841), with an estimated regression coefficient of approximately 0.0722 units of biomass per unit of N uptake. A R^2^ of about 0.9841 means that approximately 98.41% of the variation in MBY is explained by TNU in these observations. This is a very strong linear association. Importantly, the relationship is not only statistically strong but also biologically plausible: treatments with higher TNU generally produced higher MBY. Furthermore, the MBY to TNU ratio among the fertilized treatments remained within a relatively narrow range (0.120–0.136), indicating limited variation in biomass production per unit of plant N uptake. These results suggest that, over the range of nitrogen uptake represented by the fertilized treatments, assuming a constant yield coefficient is a reasonable simplification. The higher ratio observed in the unfertilized control suggests that the assumption should preferably be restricted to the fertilized/normal N-uptake range rather than extrapolated to severe N limitation. Similarly, across all treatments, MBY increased linearly with TPU, with a coefficient of determination (R^2^) of approximately 0.9549 and a regression slope of 0.1165 (Fig 4). The relatively consistent MBY to TPU ratios among the fertilized treatments further indicate that biomass production per unit of plant P uptake varied within a comparatively narrow range. Therefore, a constant yield coefficient can reasonably be assumed over the range of TPU represented by the fertilized treatments. However, the higher MBY to TPU ratio observed in the unfertilized control suggests that this assumption should not necessarily be extrapolated to severe N-limitation conditions.

For TKU (total K uptake) versus MBY, the relationship is even stronger than the previous TPU–MBY relationship (Fig 4). The R^2^ = 0.9804 is strong evidence that biomass yield and total K uptake follow an approximately linear relationship. Thus, about 98.04% of the variation in biomass yield is explained by TKU. The relatively small variation in the MBY/TKU ratio (0.0667–0.0734), indicating limited variation in biomass production per unit of K uptake among the fertilized treatments also supports the assumption that the yield coefficient can be treated as approximately constant. Therefore, a constant K yield coefficient is considered a reasonable approximation over the range of TK uptake represented by the fertilized treatments. The somewhat higher ratio in the control treatment indicates that this assumption should preferably be applied within the normal/fertilized K-uptake range rather than extrapolated to severe nutrient limitation. TKU gives very strong support for the constant-coefficient assumption, with R^2^ ≈ 0.98.

#### 3.5.3. *Nutrient requirement and response yard stick.*

**Fig 5:**
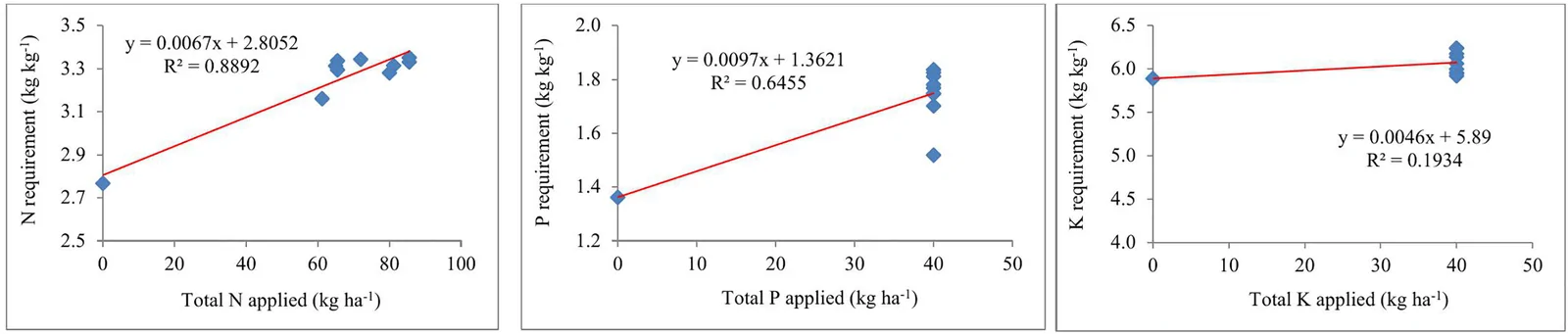
Linear regression line showing relationship between nutrient requirement and total nutrient applied (NPK) in mustard.

**Fig 6:**
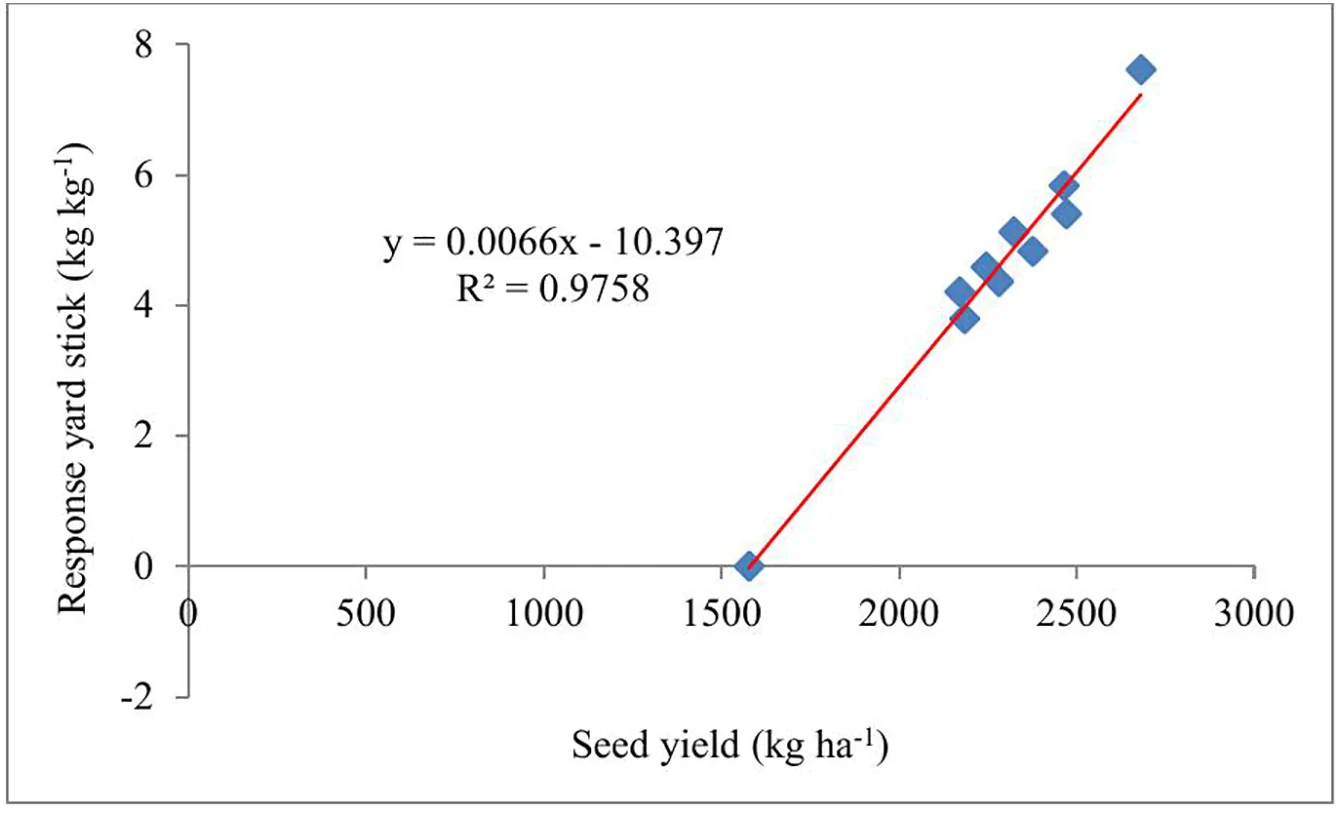
Linear regression line showing relationship between seed yield and response yard stick in mustard.

Compared with other nitrogen application strategies, GS-guided N management required less quantity of nitrogen to produce one kilogram of seed (3.31 kg kg^-1^) followed by RDN_75_ + 2% Urea FS and RDF. Whereas, P needed more in RDF and RDN_100_ + 2% NCU FS (1.84 & 1.83 kg kg^-1^) in order to generate a kilogram of seed in comparison to GS and LCC (Fig 5). Alike N, the K required to produce a kilogram of seed was again found lower under GS guided N module (5.95 kg kg^-1^) followed by RDF. Remaining other treatments at both N levels (RDN_100_ & RDN_75_) required higher amount of K for yielding per kilogram of seed. However, these values were found lower under control where we did not apply any nutrients (2.77, 1.36 & 5.70 kg kg^−1^). The yield differences between treated and untreated plots were divided by the total amount of nutrients supplied to determine the response yardstick (RYS) for each treatment. The values of RYS were ranged between 3.79 (as lowest in control) to 7.61 (highest in GS). The RYS showed substantial linear relationship with seed yield (Fig 6). The relationship revealed a strong association, with RYS explaining 97.58% of the variation in seed yield (R^2^ = 0.9758).

### 3.6. *CPK and CPE*

**Fig 7:**
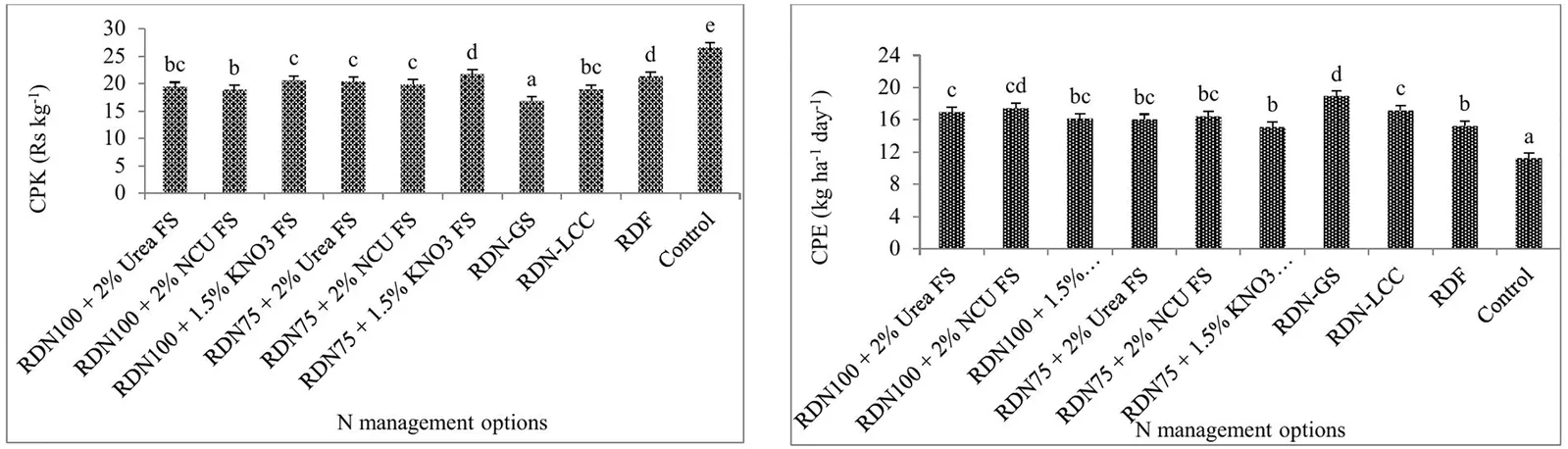
Effect of N management options on CPK (Rs kg^−1^) and CPE (kg ha^−1^ day^−1^) in mustard production (Mean data of four years).

The mean data from four years study (Fig 7) exhibited lowest cost of production per kilogram of seed (CPK) was Rs 16.8 kg^−1^ with highest per day yield, i.e., CPE (crop production efficiency) of 18.9 kg ha^−1^ day^−1^ was accomplished on administration of N fertilizer precisely through sensor based GreenSeeker device. Comparable results were recorded under LCC-based nitrogen application. The per kilogram seed production cost of mustard was significantly reduced by 21.13% on application of N through GS as against RDF followed by RDN_100_ + 2% NCU FS and LCC which did not differ significantly from each other. Further, the maximum CPK was attained under control where no fertilizer application was made. Correspondingly, about 24.35% more production per day (CPE) was achieved on application of nitrogen through GS and 14.5 & 12.6%, respectively more by adoption of RDN_100_ + 2% NCU FS and LCC over recommended practice. Amongst all treatments, the control had more CPK value of Rs 26.63 kg^-1^ with lesser CPE of 11.22 kg ha^−1^ day^−1^. In foliar sprays, RDN_100_ was comp up with marginally better results than RDN_75_ in terms of both CPK and CPE.

### 3.7. *Yield variation over RDF*

**Fig 8:**
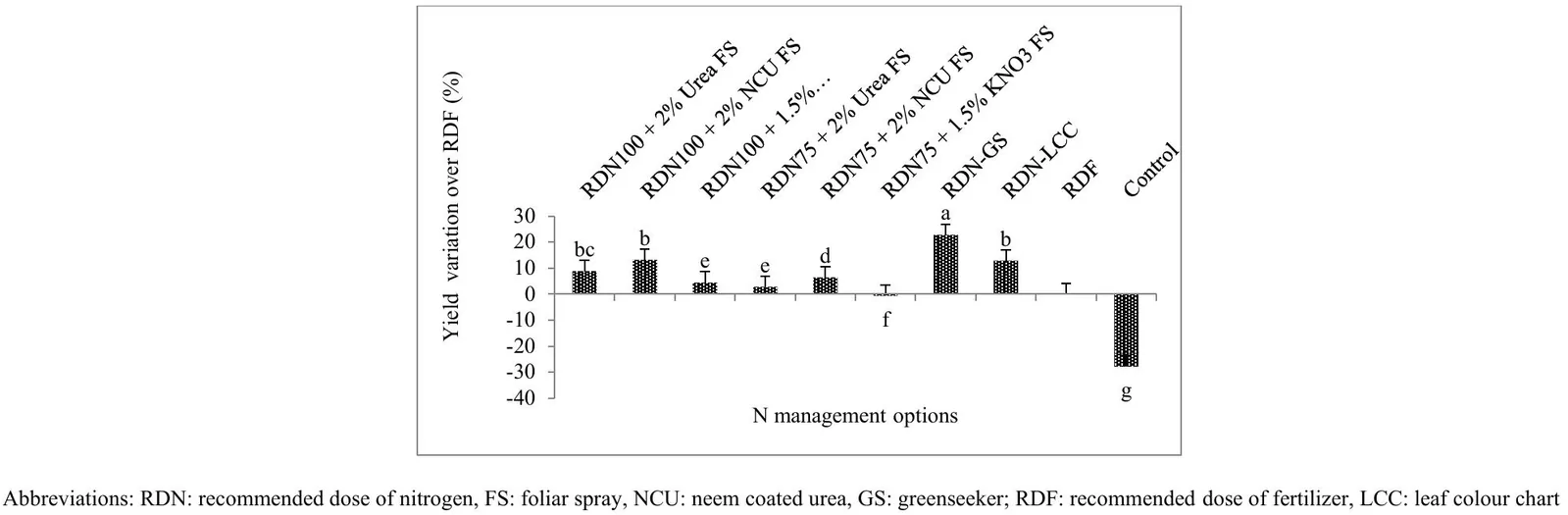
Yield variation over recommended practice (RDF) under various N management options in mustard; *(+)ve values indicates increase (more requirement) and (-)ve values shows reduction (less requirement).

**Fig 9:**
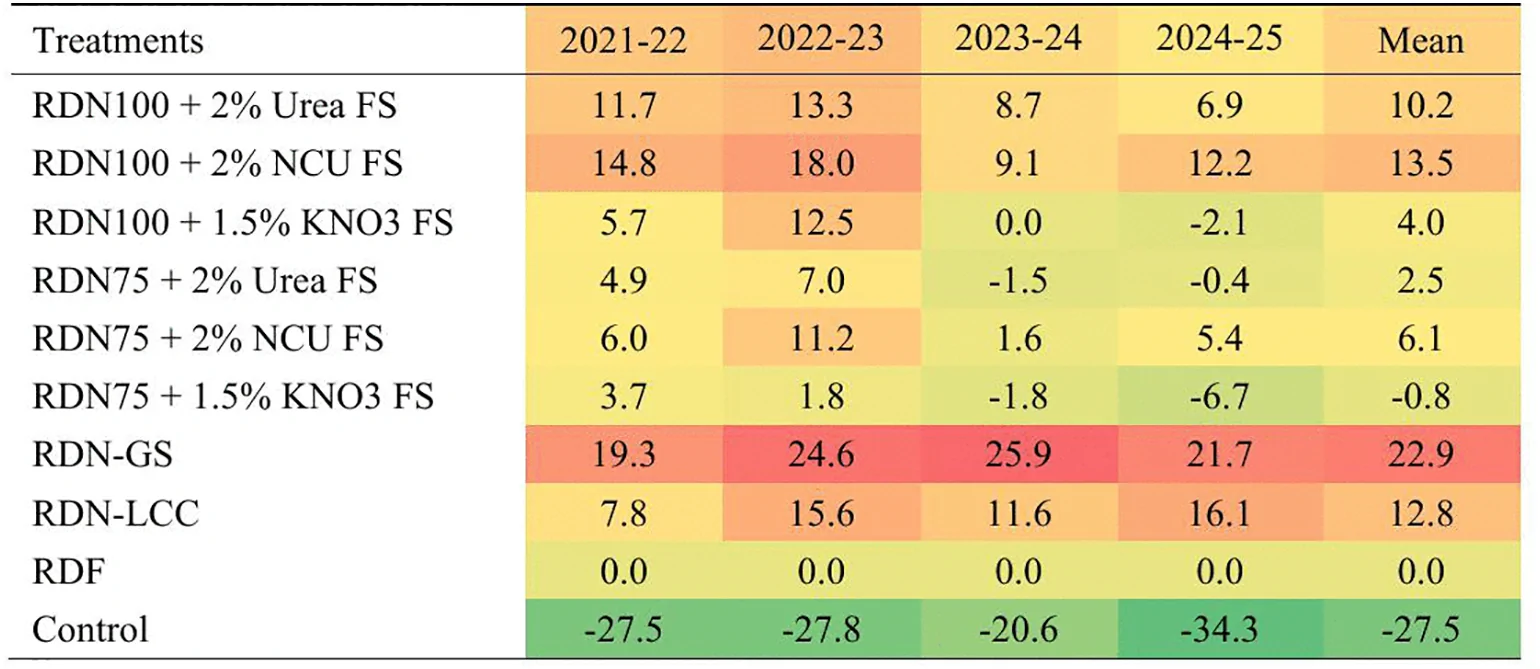
Heat map visualization of yield variation over the years under various nitrogen management practices in mustard.

Nitrogen application exerted a significant influence on seed yield under diverse N management modules. Data on seed yield (Figs 8–9) illustrated that maximum per cent reduction over RDF (−27.5%) was observed under control in comparison to all other treatments, whereas, in conversely, GS sensor guided N management significantly registered for highest seed yield increase (22.7%) followed by RDN_100_ + 2% NCU FS (13.1%) and LCC (12.8%) at par to RDN_100_ + 2% Urea FS. Non-significant improvement was recorded at RDN_75_ with N foliar applications with respect to seed yield. A linear mixed model with a random intercept for cluster was fitted to 40 observations of seed yield (kg/ha) nested within 4 clusters. The intraclass correlation was ICC = 0.42, indicating that 42.2% of the variance lay between clusters. The between-cluster variance was significantly greater than zero, F(3, 36) = 8.46, p < .001. The fixed intercept was 2277.13 (SE = 137.20); residual variance was 90796.23 and between-cluster variance was 66218.96 (AIC = 583.7). These results justify the multilevel approach and show that cluster identity meaningfully shapes the outcome.

### 3.8. *Annualized performance of nutrient-management treatments*

Considerable year-to-year variation was observed in NDVI, seed yield and nutrient uptake. Across treatments, mean NDVI was highest in 2022–23 (0.785), followed by 2021–22 (0.712), 2023–24 (0.694) and 2024–25 (0.615). Similarly, mean seed yield was highest in 2022–23 (2557.7 kg ha ^−^ ¹) and lowest in 2023–24 (1919.2 kg ha ^−^ ¹). Total N, P and K uptake followed broadly similar yearly trends, with the highest mean values recorded during 2022–23. Among the nutrient-management treatments, RDN-GS consistently recorded the highest NDVI and seed yield across all four years, with four-year mean values of 0.753 and 2681 kg ha ^−^ ¹, respectively. It also recorded the highest mean TNU (88.84 kg ha ^−^ ¹), TPU (48.56 kg ha ^−^ ¹) and TKU (159.45 kg ha ^−^ ¹). Notably, RDN-GS achieved these values with only 65 kg N ha ^−^ ¹, compared with 85.52 kg N ha ^−^ ¹ under RDN_100_ + 2% NCU FS. Thus, RDN-GS combined lower N input with superior crop growth, seed yield and nutrient accumulation. The consistency of its superior performance across the four years suggests a relatively stable treatment response.

### 3.9. *Year × N interaction (*nitrogen management strategies)

The data revealed a clear variation in the response of the parameter across different years and N management treatments (Table 5). The interaction effect of year × nitrogen management strategies revealed considerable variation among the treatment combinations for the seed yield. The mean values ranged from 1490 kg ha^−1^ in control in 2023−24 to (Y_3_N_10_) to 2962 kg ha^−1^ in 2022−23 with RDN-GS. The highest response was recorded under the combination of year 2022−23 with N management method RDN-GS (2962 kg ha^−1^), followed by RDN_100_ + 2% NCU FS in the year 2022−23(2804 kg ha^−1^), RDN-GS in 2021−22 (2756 kg ha^−1^) and RDN-LCC in 2022−23 (2747 kg ha^−1^), whereas the lowest response was observed under control in 2023−24 (1490 kg ha^−1^). The interaction effect was statistically significant at the 5 per cent level, with a critical difference (CD) of 303.567, standard error of difference [SE(d)] of 152.186 and standard error of mean [SE(m)] of 107.612. The difference between the highest and lowest treatment combinations was 1471.64, which was substantially greater than the critical difference, indicating a significant difference between these combinations. Overall, the results demonstrate that the response to different nitrogen management methods varied across years, indicating a significant year × nitrogen management interaction. Among the combinations evaluated, RDN-GS in 2022−23 was the most effective, while control in 2023−24 recorded the lowest response.

**Table 5:** Interaction effect of year and nitrogen management strategies (Y x N) on seed yield of mustard.

Year	N1	N2	N3	N4	N5	N6	N7	N8	N9	N10	Mean
2021−22	2581^bc^	2653^b^	2442^cd^	2424^cd^	2449^cd^	2395^cd^	2756^ab^	2490^c^	2310^de^	1676^h^	2418
2022−23	2693^ab^	2804^ab^	2673^ab^	2542^bc^	2644^b^	2419^cd^	2962^a^	2747^ab^	2377^d^	1716^h^	2558
2023−24	1908^fg^	1990^f^	1876^fg^	1847^g^	1906^fg^	1843^g^	2362^d^	2094^ef^	1877^fg^	1490^i^	1919
2024−25	2323^de^	2286^ab^	2402^cd^	2174^e^	2165^e^	2234^de^	2205^de^	2040^ef^	2644^b^	1428^bc^	2190
Mean N	2376	2433	2348	2247	2291	2223	2571	2343	2302	1577	

SE(m±) (Y xN) 107.612

LSD = (0.05) (Y xN) 303.567

Abbreviations: N1: RDN_100_ (50% N basal + 50% N TD) + 2% Urea FS, N2: RDN_100_ (50% N basal + 50% N TD) + 2% NCU FS, N3: RDN_100_ (50% N basal + 50% N TD) + 1.5% KNO_3_ FS, N4: RDN_75_ (50% N basal + 25% N TD) + 2% Urea FS, N5: RDN_75_ (50% N basal + 25% N TD) + 2% NCU FS, N6: RDN_75_ (50% N basal + 25% N TD) + 1.5% KNO_3_ FS, N7: RDN – Green seeker, N8: RDN – LCC (leaf colour chart), N9: RDF (recommended dose of fertilizer), N10: control.

*Means followed by the same superscript letter are not significantly different at P = 0.05.

Although the Year × N interaction was non-significant for NDVI, N applied and total nutrient uptake, the individual combinations not compared for statistical significance whereas the main effects of Year and N showed significant differences. The interpretation should therefore focus on the significant main effects of Year and N for total nutrient uptake (Table 6–7). For TNU, the year effect was significant. The highest mean value was recorded in Y2 (2022–23) at 83.277^a^, followed by Y4 (2024–25) at 78.067^b^. Y1 recorded 69.439^c^, while the lowest value was observed in Y3 (2023–24) at 66.822^d^. All four years differed significantly from one another. Among the N treatments, N7 recorded the highest mean value (88.855^a^) and was significantly superior to the other treatments. N2 (82.835^b^) and N8 (82.386^b^) were statistically at par. N1 (79.148^bc^) was intermediate. N3 (75.628^c^), N4 (73.934^c^) and N5 (77.513^c^) were statistically similar. N9 recorded 71.384^d^, N6 68.632^e^, while N10 recorded the lowest mean (43.696^f^). The highest individual numerical value was recorded under Y2 × N7 (99.443), followed by Y2 × N2 (93.547) and Y4 × N7 (93.797). The lowest value was observed under Y3 × N10 (38.757).For TPU, the Year effect was significant. The highest mean value was recorded in Y2 (2022–23) at 44.285^a^, followed by Y4 (2024–25) at 42.002^b^. Y3 (2023–24) recorded 36.327^c^, while the lowest value was observed in Y1 (2021–22) at 34.533^d^. All four years differed significantly from each other. Among the N treatments, N7 recorded the highest mean value (48.523^a^) and was significantly superior to all other N treatments. N2 (45.144^b^), N8 (43.898^b^), N1 (42.014^b^), N5 (40.637^b^), N9 (40.122^b^), N4 (39.245^b^) and N3 (38.852^b^) were statistically at par. N6 recorded a significantly lower value (32.959^c^), whereas N10 recorded the lowest value (21.473^d^). The highest individual numerical value was recorded under Y2 × N7 (55.007), followed by Y4 × N7 (51.670) and Y2 × N2 (51.417). The lowest value was observed under Y4 × N10 (21.327). Similarly, for TKU, the years, Y2 (2022–23) recorded the highest mean value (150.912), which was statistically at par with Y4 (2024–25; 149.778). Y3 (2023–24) recorded an intermediate value of 129.418, whereas the lowest mean was observed in Y1 (2021–22; 120.871). Among the N treatments, N7 recorded the highest mean value (159.448) and was significantly superior to most of the other treatments. N2 recorded the second-highest mean (152.593), while N8 recorded 147.811. The lowest mean value was recorded with N10 (89.938), indicating a markedly lower response under N10. The highest individual value was observed with Y2 × N7 (178.260), followed by Y2 × N2 (169.637) and Y4 × N7 (173.780). The lowest value was recorded with Y1 × N10 (74.017).

**Table 6:** Treatment means of main effect of different years on total nutrient uptake (N, P and K) in mustard.

Year	TNU	TPU	TKU
2021−22	69.439^c^	34.533^d^	120.871^c^
2022−23	83.277^a^	44.285^a^	150.912^a^
2023−24	66.822^d^	36.327^c^	129.418^b^
2024−25	78.067^b^	42.002^b^	149.778^a^
SE(m±)	0.852	0.517	1.967
LSD = (0.05)	2.403	1.458	5.549

Abbreviations: TNU: total nitrogen uptake, TPU: total phosphorus uptake, TKU: total potassium uptake.

*Means followed by the same superscript letter are not significantly different at P = 0.05.

**Table 7:** Treatment means of main effect of different nitrogen management strategies in mustard.

Total uptake	N1	N2	N3	N4	N5	N6	N7	N8	N9	N10	SE(m±)	LSD = (0.05)
TNU	79.148^bc^	82.835^b^	75.628^c^	73.934^c^	77.513^c^	68.632^e^	88.855^a^	82.386^b^	71.384^d^	43.696^f^	1.347	3.799
TPU	42.014^b^	45.144^b^	38.852^b^	39.245^b^	40.637^b^	32.959^c^	48.523^a^	43.898^b^	40.122^b^	21.473^d^	0.817	2.305
TKU	148.323^bc^	152.593^ab^	139.933^cd^	140.005^cd^	140.818^cd^	129.303^e^	159.448^a^	147.811^bc^	129.277^e^	89.938^f^	3.110	8.774

Abbreviations: TNU: total nitrogen uptake, TPU: total phosphorus uptake, TKU: total potassium uptake, N1: RDN_100_ (50% N basal + 50% N TD) + 2% Urea FS, N2: RDN_100_ (50% N basal + 50% N TD) + 2% NCU FS, N3: RDN_100_ (50% N basal + 50% N TD) + 1.5% KNO_3_ FS, N4: RDN_75_ (50% N basal + 25% N TD) + 2% Urea FS, N5: RDN_75_ (50% N basal + 25% N TD) + 2% NCU FS, N6: RDN_75_ (50% N basal + 25% N TD) + 1.5% KNO_3_ FS, N7: RDN – Green seeker, N8: RDN – LCC (leaf colour chart), N9: RDF (recommended dose of fertilizer), N10: control.

*Means followed by the same superscript letter are not significantly different at P = 0.05.

### 3.10. *Per cent change in SOC*

**Fig 10:**
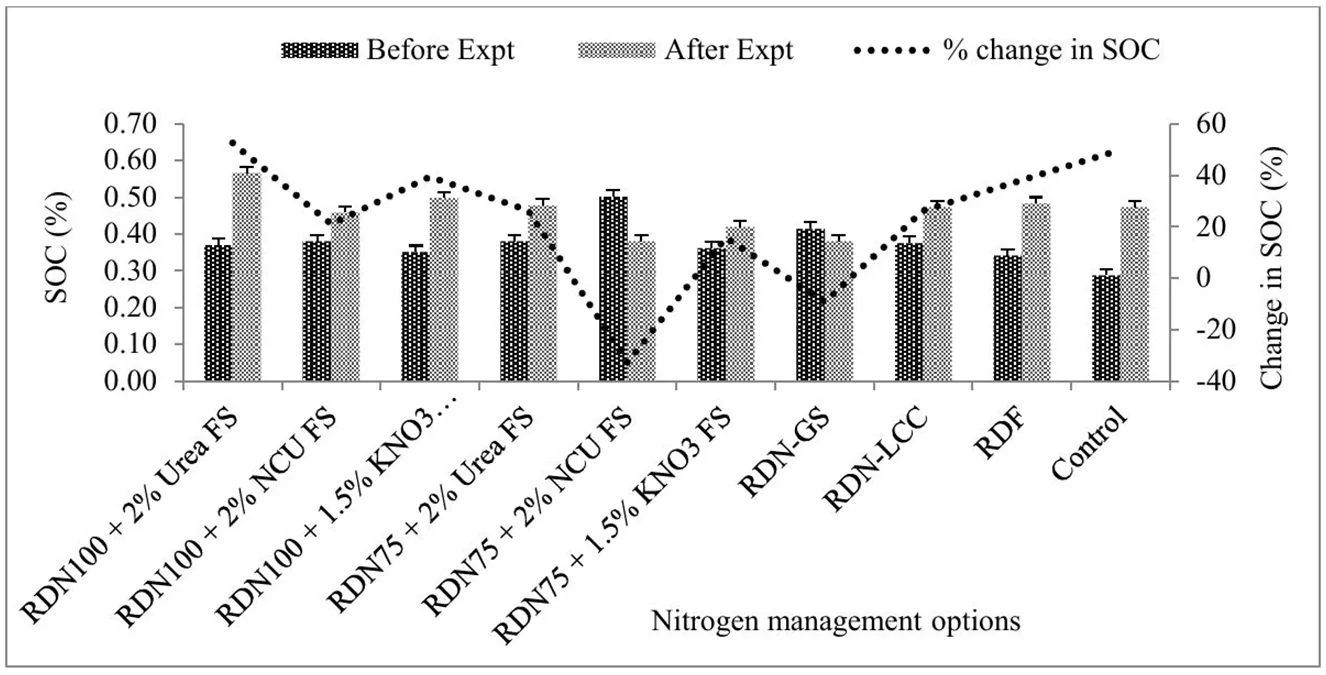
Per cent change in soil organic carbon (SOC) content over recommended practice (RDF) under various N management options in mustard.

An increase in soil organic carbon (SOC) content over the initial value was observed in all nitrogen management treatments, except under RDN_75_ + 2% NCU FS and GS-based nitrogen application (Fig 10). The relative changes in SOC (%) before and after experimentation were positive under all treatment excluding above mentioned these two N management options. The per cent enrichment in SOC content was found superior in RDN_100_ + 2% Urea FS (52.6%) followed by RDN_100_ + 1.5% KNO_3_ FS (39.4%) which were statistically comparable with recommended practices, i.e., RDF (38.1%). Foliar application of NCU at both N levels (RDN_100_ & RDN_75_) was less performer in relation to enhancement of SOC content over other treatments. The GS guided N application also depreciated SOC content from initial value due to reduced amount of N application, whereas, remaining treatments showed increasing trend of SOC content at harvest after four years of experimentation.

### 3.11. *Carbon footprint*

The carbon emissions associated with different nitrogen management methods are presented in Table 8. Among all agricultural inputs, fertilizers contributed the largest share of total carbon emissions, accounting for 45.6–52.3%, whereas the contributions from other inputs were comparatively lower across all treatments. Total CO_2_ emissions were higher under the 100% recommended dose of nitrogen (RDN) with foliar nitrogen application than under the 75% RDN treatment. In contrast, the GreenSeeker (GS)-based nitrogen application reduced CO_2_ emissions by 11.23% compared with the recommended fertilizer dose (RDF). Similarly, N_2_O emissions were lowest under GS-based nitrogen management, showing an 18.9% reduction compared with RDF. The yield-scaled carbon footprint (CFy) ranged from 111.2 to 139.0 kg CO_2_-eq t ^−^ ¹ of mustard biological yield across the nitrogen management treatments. The lowest CFy was recorded under the control treatment, primarily due to the absence of nitrogen fertilizer inputs, despite its lower crop yield.

**Table 8:** Effect on carbon footprint under diverse nitrogen management modules in *Brassica juncea* L. (Mean data of four years).

Treatments	Total CO_2_ (kg CO_2_-e ha^−1^)	N_2_O (kg CO_2_-e ha^−1^)	GWP (kg CO_2_-e ha^−1^)	CFy (kg CO_2_-e t^−1^)	% contribution from fertilizers
RDN_100_ (50% N basal + 50% N TD) + 2% Urea FS	944	400	1344	131.0	45.6
RDN_100_ (50% N basal + 50% N TD) + 2% NCU FS	944	400	1344	129.5	45.6
RDN_100_ (50% N basal + 50% N TD) + 1.5% KNO_3_ FS	922	380	1302	131.3	46.7
RDN_75_ (50% N basal + 25% N TD) + 2% Urea FS	844	307	1151	117.5	51.0
RDN_75_ (50% N basal + 25% N TD) + 2% NCU FS	844	307	1151	118.5	51.0
RDN_75_ (50% N basal + 25% N TD) + 1.5% KNO_3_ FS	823	286	1109	119.4	52.3
RDN – Green seeker	842	304	1146	111.2	51.1
RDN – LCC	877	337	1214	126.6	49.1
RDF	916	375	1291	139.0	47.0
Control	519	0	519	74.0	–

Abbreviations: RDN: recommended dose of nitrogen, TD: top dressing; FS: foliar spray, NCU: neem coated urea, RDF: recommended dose of fertilizer, LCC: leaf colour chart, CO_2_: carbon dioxide, N_2_O: nitrogen oxide, GWP: global warming potential; CFy: yield-scaled carbon footprint

### 3.12. *Linear regression between NDVI and seed yield*

**Fig 11:**
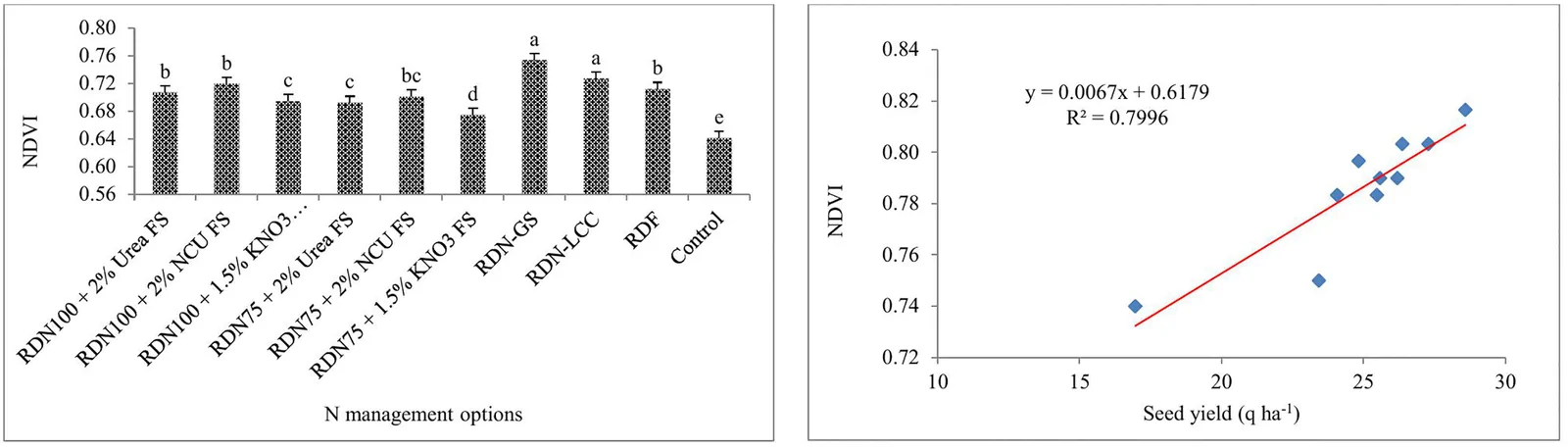
Relationship exhibiting effect of N management options on NDVI and seed yield through Linear regression line in mustard (Mean data of four years).

The data in the Fig 11 showed the influence of various N management strategies on normalized difference vegetation index (NDVI), is a widely-used metrics for quantifying the health and density of vegetation using sensor data derived from the difference between near-infrared and red light reflectance. The N administration using sensor based devises, i.e., GreenSeeker (GS) registered higher value of NDVI followed by LCC based N application then remaining other treatments. Over the recommended practice (RDF), the per cent increment in NDVI value was 5.63% greater under GS guided N application. The higher value of the NDVI in crop plants indicates their leafy greenness which is due to leaf N content. Higher NDVI means, higher chlorophyll (N content) in the leaf that shows healthiness and vigourness of the plant. The control treatment has lower NDVI value among all the treatment as it does not received N application. Among the foliar sprays of N applied via various means, NCU @ 2% was performed better in comparison with urea at the same rate or KNO_3_ at 1.5% at RDN_100_ level.

A pronounced year-to-year variation in NDVI was observed across the nutrient-management treatments. The mean NDVI across treatments was highest during 2022–23 (0.785), followed by 2021–22 (0.712), 2023–24 (0.694), and 2024–25 (0.615). Among the treatments, RDN-GS consistently recorded the highest NDVI across all four years (0.79, 0.82, 0.74 and 0.66, respectively), with a four-year mean of 0.753, whereas the control recorded the lowest values (0.63, 0.74, 0.62 and 0.57; mean 0.640). The N application ranged from 61.17 to 85.52 kg N ha ^−^ ¹ among the fertilized treatments, while RDN-GS achieved the highest mean NDVI (0.753) with 65 kg N ha ^−^ ¹. The treatment rankings were broadly consistent across years, although the magnitude of treatment differences varied between seasons, indicating a possible year × treatment interaction. RDN-GS produced the highest mean NDVI with substantially less applied N than the RDN100 treatments. This is potentially an important finding because it points toward improved N-use efficiency rather than simply greater N application.

## 4. Discussion

Nitrogen fertilizers are integral to numerous physiological processes involved in growth and development of the plants, playing crucial role in sustainable food production. In the initial stages of growth, crop nitrogen demand is typically met by native soil N and nutrient reserves within the seed, which are sufficient to support germination and early seedling development. However, as the plant progresses to later stages of growth, when nitrogen demand peaks, the importance of timely applied nitrogen either through soil or foliar becomes more evident. Nevertheless, the excessive application of N fertilizers often surpassing recommended rates is a common practice to fulfil plant nutrient requirements. Conversely, inadequate soil nitrogen availability adversely affects dry biomass accumulation at later stages of plant growth. Large portion of nitrogen (around 50%) applied as a basal dose during early crop stages when root systems are still underdeveloped may result in significant N losses. These losses reduce fertilizer use efficiency and also lead to environmental degradation [34]. Earlier studies revealed that excessive basal N application during early vegetative stages elevates soil ammonia levels, which further intensifies nitrogen loss [35–37]. In-season monitoring of N uptake and yield is essential for setting up strategies to optimize N fertilization and reduce the environmental risks associated with fertilizer N use. Inadequate prediction of yield and N uptake when using site-specific N management strategies may result in over- or under-recommendation of N fertilizer than the required [38]. In fact, portable sensors have opened up a new approach to acquire crop growth information in rapidly and in a non-invasive manner. The losses of N, however, can be reduced by adopting an innovative N management practice in real time using GreenSeeker crop sensor besides enhancing crop productivity and nitrogen use efficiency. NDVI as measured by GreenSeeker sensor has been shown to be useful in acquiring information such as potential yield, photosynthetic efficiency and N status [39]. Application of N based on our developed N estimation rate chart (NERC) was significantly enhanced yield and yield attributing characteristics. Four years mean data revealed that NDVI readings obtained through GreenSeeker for determining N prerequisite of the plants followed by its application on the basis of NDVI-nitrogen estimation rate chart (NERC) showed utmost improvement in siliquae number per plant (13.4%), seed per siliquae (5.73%) and 1000 seed weight (3.67%) over recommended practices (RDF). These improvements are likely due to better N availability in the soil, which amplified nutrient acquisition. Moreover, synchronizing soil N supply with crop demand diminishes nitrogen forfeiture and improves its use efficiencies. Concordant findings have been reported in previous studies [13,40–42].

As a component of total dry biomass partitioned into seeds, yield is regulated by multiple bio-physiological processes (Singh et al. 2022), reflecting a strong source-sink relationship has proven effective in improving both seed yield and NUE in mustard. Among the tested strategies, GS-based N management outperformed other methods in enhancing seed yield and nitrogen use efficiency (NUE) in *Brassica juncea* (L.). Over a four-year average, GreenSeeker-based multi-split N application increased yield by 22.7% and 70.0%, respectively over suggested dosage of fertilizers (RDF) and control. This enhancement can be ascribed to increased soil nutrient availability and better synchronization between crop N demand and soil supply, which enriched soil fertility and created favourable conditions for plant growth. Additionally, enhanced photosynthate allocation to both vegetative and reproductive organs during later growth stages contributed to better overall plant vigour [42–44]. These enhancements resulted from sufficient nutrient availability, which promoted plant growth and facilitated better assimilate translocation from source to sink [45]. Our findings align with those reported by Meena et al. [46–48]. Applying nitrogen in multiple splits effectively mitigates nitrogen losses commonly caused by volatilization, leaching, denitrification, and other pathways. Studies have shown that up to 50% of applied nitrogen can be lost through these mechanisms. Reducing such losses ensures a greater proportion of nitrogen is absorbed by the crop, thereby enhancing overall crop growth and development.

Correspondingly, Singh et al. [49] observed that N application made through GreenSeeker crop sensor significantly improved yield in irrigated wheat across the Indo-Gangetic plains of South Asia. Synchronizing nitrogen application with crop’s peak demand period minimizes losses through the soil–plant system which turn to improved NUE. Jiang et al. [50] reported that N application in potato made through Nutrient Expert (NE) results 7% yield increase while reducing nitrogen input by 21 percent. Moreover, nitrogen losses and nitrogen balance were reduced by 31% and 48%, respectively. The NE enabled nitrogen saving of 19 kg/ha without any yield or profit reduction, while decreasing nitrogen losses and balance by 13% and 27%, respectively. Previous studies have further demonstrated that lower, well-timed nitrogen doses can be more effective in boosting yield than higher, unregulated applications [51]. These findings align with the present results, confirming that tools such as NE and GreenSeeker significantly augmented yield traits through effective nitrogen management [52,53]. Late-season N splitting in maize increased grain yield, total dry biomass, NUE metrics *viz*. agronomic efficiency (AE), partial factor productivity (PFP), physiological efficiency (PE), and recovery efficiency (RE) over conventional N application practices [54]. Quantitatively, these results are broadly consistent with previous GreenSeeker/photosensor investigations, although the metrics are not directly interchangeable. This is in agreement with Ali et al. [55] who revealed that GreenSeeker optical sensor can better predict N uptake and grain yield in wheat at the jointing stage as it could explain 69% 9 (R^2^ ≈ 0.685) and 61% (R^2^ ≈ 0.606) variability as compared to measurements atLeaf chlorophyll meter, which explained 55% and 53% variations in N uptake and grain yield, respectively. Other wheat experiments in the Indo-Gangetic region reported R^2^ values of 0.61 and 0.76 for relationships between in-season estimated yield and potential yield at Feekes 5–6 and Feekes 7–8, respectively [44]. Similarly, a cotton study obtained an R^2^ of 0.80 between GreenSeeker NDVI and lint yield [56]. The agronomic consequences reported in earlier studies also support the mechanism represented by the present normalized-rate curve. In wheat, GreenSeeker-guided N application has achieved grain yields comparable to blanket applications while reducing N inputs; one study reported 17–24 kg N ha ^−^ ¹ savings, up to 59.5% N recovery, and approximately 2.5% higher grain yield than a 150 kg N ha ^−^ ¹ conventional treatment [57]. Another wheat study found GreenSeeker-guided applications of approximately 123–133 kg N ha ^−^ ¹**,** compared with the conventional 150 kg N ha ^−^ ¹ recommendation, indicating a potential saving of roughly 11–18% N without compromising crop quality. In rice, sensor-guided N management has similarly been reported to improve N-use efficiency by more than 12% while maintaining yields comparable with general fertilizer recommendations [40].

Besides soil applications, nitrogen application on foliage either as an alone treatment or in combined with other nutrients, has demonstrated yield benefits, especially applied at critical growth stages. Foliar feeding enables direct nutrient absorption through stomata and internal transport *via* plasmodesmata [58]. Among the tested foliar treatments, a 2% neem-coated urea (NCU) solution proved more effective than 2% urea or 1.5% potassium nitrate (KNO_3_). Moreover, greater economic yield and lesser production cost attributed to the maximum cost per kilogram (CPK). Further, yield variability amongst treatments with same growing period (crop duration) resulted in greater crop production efficiency as recorded under GS and LCC based N application compared to the recommended practices. The results of the present investigation were supported by the findings of Maheshwara et al. [59].

Improvements in soil fertility parameters, particularly regarding the accessibility of nitrogen (N), phosphorus (P), and potassium (K), can be ascribed to increased nutrient uptake by plants [60,61]. Additionally, synchronisation of N supply with crop demand can minimize nutrient losses while promoting more efficient nutrient absorption. This may be ascribed to the balanced and sustained nutrient supply provided through sensor guided real time N management supports prolonged leaf greenness, increased photosynthetic efficiency, dry biomass production facilitates efficient translocation of photosynthates to sink tissues. More recent work further supports the quantitative reliability of optical sensing. In spring maize, a GreenSeeker-based sufficiency-index approach increased total N uptake by approximately 10.7–15.0% relative to soil-test-based N management at 120 kg N ha. These results indicate that the response curves in the present figure are not merely empirical graphical relationships; they represent a broader sensor-based management mechanism in which spectral differences → NDVI → crop N/vigor status → normalized fertilizer requirement. One important qualification is that the 30% normalized rate obtained from the present graph should not be directly compared with the R^2^ values reported in previous studies. The former is an algorithmic fertilizer-response output, whereas R^2^ measures predictive association between sensor measurements and crop variables. A stronger quantitative validation of the present approach would therefore compare the calculated normalized rates with actual N requirement, N uptake, grain yield, agronomic N-use efficiency, and percentage N saving. On the basis of previous studies, a successful implementation would ideally maintain yield while reducing N input by roughly 10–20% or more, with improvements in N recovery/use efficiency. In a study of Singh and Singh [49], results showed that N application using SPAD based on sufficiency index of 0.95 and GreenSeeker based on 0.90 sufficiency index criteria not only produced significantly higher yields but also increased total N uptake by 10.7% to 15.0% than soil-test based fertilizer N recommendation with fertilizer N dose of 120 kg N ha^−1^. Significantly higher N recovery and agronomic efficiencies were observed when N was applied using SPAD and GreenSeeker based sufficiency index approaches.

In the present investigation, the different nitrogen management modules exhibited distinct carbon dynamics. As expected, higher levels of nutrient application resulted in greater carbon inputs due to the increased use of agricultural inputs. The results indicated that the application of 100% of the recommended dose of nitrogen (RDN) supplemented with foliar nitrogen through urea, neem-coated urea (NCU), or potassium nitrate (KNO_3_) was the most carbon-intensive nitrogen management strategy, resulting in a 2.96% higher carbon input than the recommended fertilizer dose (RDF). This increase can be attributed to the combined effects of additional fertilizer inputs and the extra labour and field operations required for foliar nutrient application. Carbon footprint is primarily associated with N dosages and has a significant impact on the crop’s ability to convert that nitrogen into yields [62]. NDVI based nitrogen management was associated with a lower estimated greenhouse-gas emission footprint by approximately 11.23% (particularly nitrous oxide emission) based on the applied emission factors in comparison to recommended nitrogen application strategy, primarily due to improved nitrogen use efficiency [63]. Based on emission-factor calculations, RDN-GS showed a lower estimated greenhouse-gas footprint than the conventional nutrient-management treatments. These values represent estimates rather than directly measured field emissions and should therefore be interpreted as indicative of potential mitigation rather than verified reductions in greenhouse-gas emissions. These findings highlight the potential of precision nutrient management to simultaneously enhance resource-use efficiency and mitigate the environmental impacts of agricultural production, thereby contributing to more sustainable crop production systems.

A limitation of the present study is that greenhouse-gas emissions were estimated using emission factors rather than measured directly in the field. Consequently, the calculated values represent potential or estimated emissions associated with fertilizer inputs and should not be interpreted as direct measurements of N_2_O or total greenhouse-gas fluxes. Generic emission factors may not adequately capture the temporal and spatial variability of emissions under different nutrient-management practices and environmental conditions. In particular, N_2_O emissions can be strongly affected by soil moisture, temperature, soil mineral N availability, rainfall or irrigation events, fertilizer source, application timing and placement, and crop growth dynamics. The present estimates therefore provide a comparative indication of the potential greenhouse-gas implications of the treatments, rather than definitive evidence of actual emission reductions. Future studies should incorporate direct measurements of N_2_O and other relevant greenhouse-gas fluxes using appropriate field-based measurement techniques to validate the estimated mitigation potential. Other limitations include the relatively limited number of experimental seasons and the dependence of treatment responses on prevailing seasonal conditions. Although four years of observations provide a useful basis for assessing treatment consistency, additional site-years and locations would strengthen the inference regarding the stability and wider applicability of the results.

**Fig 12:**
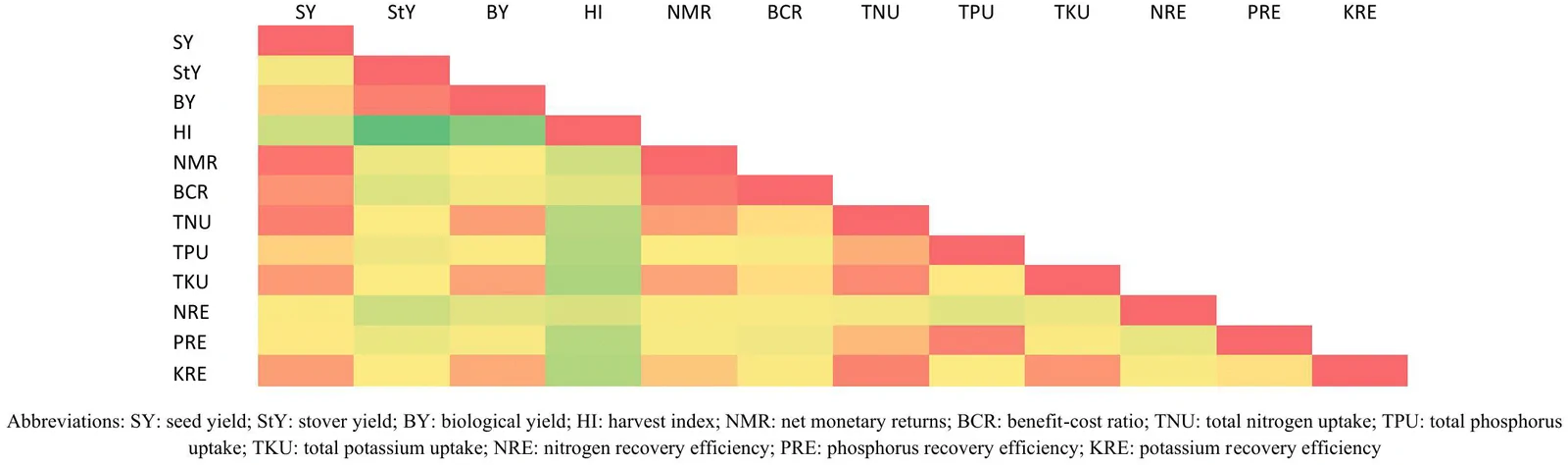
Correlation analyses of productivity, profitability and nutrient indices to different nitrogen management practices.

**Fig 13:**
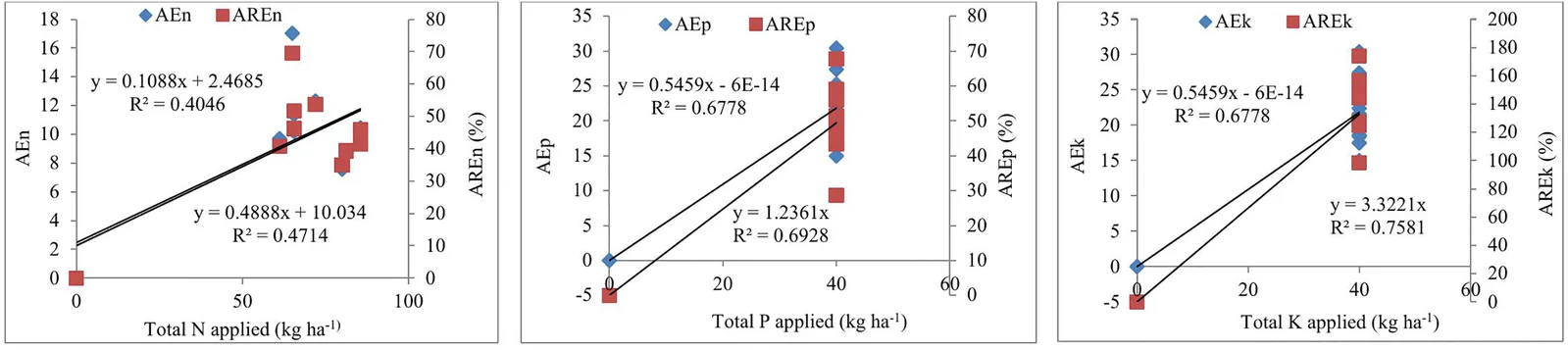
Relationship between agronomic efficiency (AE), agronomic recovery efficiency (ARE) of N, P and K with total nutrient applied in mustard.

A notably positive linear association was shown in the regression analysis between total NPK absorption and the biological yield of mustard (Fig 4). The constant yield coefficient assumption was supported by the measured plant nitrogen data because biomass accumulation showed an approximately proportional relationship with plant nitrogen uptake. The estimated biomass to N ratios remained relatively stable across sampling periods, with no systematic trend in the residuals. Thus, although some variation in nitrogen use efficiency occurred, it was sufficiently small that a constant yield coefficient provided an adequate representation of the measured plant response over the experimental range. The mean biological yield (seed + stover) of crop exhibited a positive relationship with total nitrogen (r^2^ = 98), total phosphorus (r^2^ = 95) and total potassium (r^2^ = 98) uptake (Table 9). Significant correlation was exists between the MBY and total nutrient uptake and productivity, profitability and nutrient indices to various nitrogen management practices (Fig 12). The linear correlation was also exhibited with N/P/K application in terms of agronomic efficiency-AE (seed yield) and agronomic recovery efficiency-ARE (nutrient recovery). These representations showed that how the crop responses changed even with constant rate (fixed) of nutrient application which was expressed through AE and ARE (Fig 13). The outcomes are in line with the findings of [64,65].

**Table 9:** Linear regression analysis between nutrient uptake and biological yield of mustard (Mean data of four years).

	Nitrogen			Phosphorus			Potassium		
	Regression	Residual	Total	Regression	Residual	Total	Regression	Residual	Total
SS	7.11	0.110	7.22	6.89	0.325	7.22	7.08	0.141	7.22
Mean of squares	7.11	0.013		6.89	0.040		7.08	0.017	
R	0.99			0.98			0.99		
R^2^	0.98			0.95			0.98		
Adjusted R^2^	0.97			0.94			0.97		

## 5. Conclusion

The present study demonstrated that NDVI-based precision nitrogen management is an effective and sustainable approach for improving nitrogen use efficiency while maintaining or enhancing crop productivity. Nitrogen management through Nitrogen Estimation Rate Chart (NERC) enabled real-time application of N according to crop requirement and target yield. The study showed that the optimized nitrogen management in real time and in right amount increased seed yield by 22.75%, while saving 18.7% N compared with the recommended practices. It also substantially improved nitrogen-use efficiency parameters, including AEN, PFPN and NRE. In addition, improved soil organic carbon and lower greenhouse-gas emission over RDF highlight the environmental benefits of precision N management. Overall, the findings support the hypothesis that increasing nitrogen use efficiency through real-time, sensor-based N management can reduce unnecessary fertilizer use without yield penalty, improve profitability, conserve soil and nutrient resources, and reduces environmental impacts, particularly N_2_O emissions. The lower greenhouse-gas values estimated for treatments receiving less N was interpreted based on emission factor rather than measured reductions in field greenhouse-gas emissions. Therefore, further studies involving direct measurement of N_2_O and other relevant greenhouse-gas fluxes, together with additional site-years, would be useful for validating the environmental implications of these nutrient-management strategies. Thus, NDVI-based N management coupled with NERC offers a promising precision-farming technology for achieving higher productivity with less N input, lower production costs and enhanced environmental sustainability.
